# Peer volunteers in an integrative pain management program for frail older adults with chronic pain: study protocol for a randomized controlled trial

**DOI:** 10.1186/1745-6215-15-205

**Published:** 2014-06-03

**Authors:** Mimi Mun Yee Tse, Paul Hong Lee, Sheung Mei Ng, Bik Kwan Tsien-Wong, Suey Shuk Yu Yeung

**Affiliations:** 1 School of Nursing, The Hong Kong Polytechnic University, Hung Hom, Kowloon, Hong Kong SAR; 2 Department of Rehabilitation Sciences, The Hong Kong Polytechnic University, Hung Hom, Kowloon, Hong Kong SAR; 3 Department of Applied Social Sciences, The Hong Kong Polytechnic University, Hung Hom, Kowloon, Hong Kong SAR

**Keywords:** protocol, peer volunteers, pain, non-pharmacological, physical activity, gerontology

## Abstract

**Background:**

Chronic pain is common among the older population. A literature review on pain management program showed that exercise, yoga, massage therapy, Tai Chi, and music therapy could significantly reduce pain. In spite of the proven benefits of pain management programs, these intervention programs were effective only in the short term, and older adults would resume their old habits. It has been suggested that interventions comprising some type of social support have great potential to increase the participation of older adults. Therefore, we propose the inclusion of peer volunteers in an integrated pain management program to relieve pain among frail older adults. This study aims to explore the effectiveness of an integrated pain management program supplemented with peer volunteers in improving pain intensity, functional mobility, physical activity, loneliness levels, happiness levels, and the use of non-pharmacological pain-relieving methods among frail older adults with chronic pain.

**Methods/Design:**

We intend to recruit 30 nursing home residents and 30 peer volunteers from the Institute of Active Ageing in Hong Kong in a group trial for an 8-week group-based integrated pain management program. There will be 16 sessions, with two 1-hour sessions each week.

The primary outcome will be pain levels, while secondary outcomes will be assessed according to functional mobility, physical activity, loneliness levels, happiness levels, the use of non-pharmacological pain-relieving methods, and through a questionnaire for volunteers.

**Discussion:**

In view of the high prevalence of chronic pain among older adults and its adverse impacts, it is important to provide older adults with tools to control their pain. We propose the use of peer volunteers to enhance the effects of an integrated pain management program. It is expected that pain can be reduced and improvements can be achieved among older adults in the areas of physical activity, functional mobility, loneliness levels, happiness levels, and the use of non-pharmacological pain relieving methods. Using these results, we will assess the need to conduct a larger study with a randomized controlled design.

**Trial registration:**

This trial was registered on 24 February 2014 at the Australian New Zealand Clinical Trials Registry (ANZCTR) with the trial number: ACTRN12614000195651

## Background

An aging population is a global phenomenon. As people get older, they become more vulnerable to age-related diseases and the resulting pain. The prevalence of chronic pain among community-dwelling older adults is high. For instance, in Hong Kong, 37 to 50% of community-dwelling older people suffer from pain [1,2]. The prevalence of pain among nursing home residents in Hong Kong may even be as high as 70% [3].

Chronic pain can have severe adverse impacts on older adults. The consequences of unrelieved chronic pain include hindered activities of daily living, depression and anxiety, decreased social interaction, impaired mobility, falls, sleep disturbance, malnutrition, and increased health care utilization and expenses [4]. These adverse effects of chronic pain may worsen pre-existing health problems, thereby increasing the burden on health care and social services.

Analgesics remain the primary method of managing chronic pain [4]. However, some older people prefer not to use pain medication and often request them only when the pain reaches an intolerable level. In a local study, 173 community-dwelling older persons were interviewed and only 47% of the participants used oral analgesic drugs to relieve their pain [5].

Due to the limited acceptance of pharmacological approaches to managing pain, an increasing number of studies are being conducted to examine the effectiveness of non-pharmacological strategies to relieve pain. In a literature review, Reid *et al.*[6] investigated the evidence on self-managed interventions for pain among older adults. A total of 27 articles were identified, including those that evaluated programs sponsored by the Arthritis Foundation and other programs such as yoga, massage therapy, Tai Chi, and music therapy. Among the reviewed studies, 96% showed positive outcomes, with a median reduction in pain scores of 23%.

Another 8-week integrated pain management program (IPMP) was carried out among Hong Kong nursing home residents [7]. A total of 535 older adults were randomized into an experimental group (n = 296) and a control group (n = 239). The participants in the control group only received regular care but not the IPMP. The participants in the experimental group received the IPMP, which included a 30-minute physical exercise program (PEP) and 30 minutes of either multisensory stimulation therapy or an arts and crafts activity each week. The PEP included exercises in muscle strengthening, stretching, and balancing, and was conducted in small groups of 10 to 15 older residents. In the multisensory session, relaxation techniques to control pain and the use of the five primary senses were taught. In the arts and crafts session, older adults made artwork such as photo albums, paper flowers, and paper fans to exercise their fine motor activities and for multisensory stimulation. Upon completing the program, the participants in the experimental group showed a significantly higher reduction in pain scores, higher happiness and life satisfaction scores, and lower scores in loneliness and depression than were observed among the control group; no significant differences were seen in the control group. A significant increase in the use of non-pharmacological methods of pain relief was also seen among the participants in the experimental group as compared to the control group.

Although the pain management program, which includes physical activity, has been shown to have health benefits and improve pain management among older adults [8], studies have found educational interventions to be effective among older adults only over the short term [9]. People who participated in these interventional programs often fell back into their old habits of inactivity after completing the program [10]. Therefore, it is important to develop interventions that can be sustained over time.

It is suggested that interventions with some type of social support, such as a ‘buddy’ system, have great potential to increase the participation of older adults in exercise and overall fitness activities [11], and will lead to greater adherence to such activities, as well as result in a higher level of enjoyment of them [12]. Social support interventions in community settings focus on changing physical activity behavior through building, strengthening, and maintaining social networks that provide supportive relationships for behavioral change. In general, these interventions involve making a ‘contract’ with companions to achieve specified levels of physical activity, or setting up walking or other groups to provide companionship and support while being physically active [11]. In a study conducted in the United States, 81 sedentary adults were randomly assigned to two 16-week group-based programs: 1) peer-delivered or 2) a ‘standard’ community-based exercise promotion intervention. No significant difference in moderate-to-vigorous physical activity (MVPA) minutes/week at week 16 was found between the two groups, but both reported significantly more MVPA minutes/week relative to baseline at week 16. Moreover, peer volunteers (PVs) enhanced the long-term maintenance of physical activity, as the active intervention group was able to maintain and slightly increase their PA behavior by the 18-month follow-up session, while the standard community intervention group began to return to their baseline levels. There was significantly more physical activity among the group with peer support relative to the standard community intervention group after 18 months [13].

The effectiveness of PVs was also studied in a fall prevention program. After a 10-week intervention program, participants (n = 52, aged 65 to 94) in the peer-led group reported a 27% decrease in falls and more weekly walking for 34 minutes compared with the control group (n = 25), who had been taught by a trained instructor and had been without peer support [14].

Our research team conducted a search of databases (PubMed and ScienceDirect) to study the application of PVs in intervention programs for older adults in Hong Kong from 1998 to the present. Four studies were identified, and they have been shown to achieve positive outcomes. However, the programs studied were those for healthy living, coping with stress, post-discharge support, and physical activity [15-18]. No pain management programs involving PVs were found. PVs were chosen over other forms of social support as the Hong Kong population is aging and people are living longer. After retirement, some older adults are still enthusiastic about serving the community. Therefore, we will select the PVs from the Institute of Active Ageing (IAA), which is dedicated to promoting active aging and the view of older adults as important contributors to society. As the IPMP has already been validated and published, and its effectiveness has been established, the effectiveness of the IPMP among older adults is well understood; however, whether this effect will be extended with the use of PVs is unknown. Therefore, in the present study we will attempt to test only the effectiveness of an IPMP that will be sustained by PVs.

The aim of this study is to determine the feasibility of PVs in an IPMP for Hong Kong older adults with chronic pain. According to the literature, pain management programs range from 4 weeks to 8 weeks. As the previous 8-week IPMP has already been validated, in this study we would also like to examine the dose-response relationship of an IPMP with PVs (that is, the possibly nonlinear association between intervention duration and pain intensity score). Our objective is to run a pilot study for a future main study with a randomized controlled design. It is hypothesized that the implementation of an IPMP supplemented with PVs for older adults with chronic pain will lead to improvements in levels of pain intensity, functional mobility, physical activity, loneliness, and happiness, and in the use of non-pharmacological pain-relieving methods.

## Method/Design

### Design

It will be a single group trial.

### Setting

The 8-week group-based IPMP will be held in one nursing home in Hong Kong.

### Samples

#### Participants

The participants will be recruited from one nursing home in Hong Kong. The home-in-charge nurse will be approached and invited to participate in the study. Older adults in the nursing home who are interested to participate will be recruited according to the inclusion criteria. The present study will be a pilot study with 30 participants. Inclusion criteria are older adults aged ≥60, who scored ≥6 in the Abbreviated Mental Test [19], have been experiencing non-malignant physical pain or discomfort either all the time or on and off for more than three months, scored ≥1 in the frailty index [20], and are able to speak and understand Cantonese. Exclusion criteria are those with cognitive impairment and a history of mental disorders, and those with cancer and currently on cancer treatment, since the present study is for older adults with non-malignant pain. As this program involves physical activities, those suffering from conditions that limit safe participation will be excluded. Examples include those who have had a fracture or undergone surgery in the past two months, those with severe chronic obstructive pulmonary disease, acute stroke, and acute myocardial infarction. The ratio of participants to PVs is 1:1. PVs who have not been paired with participants will be responsible for making overall observations during the sessions. Participants and PVs will also be matched according to gender and dialect. Since it is essential that actions be repeated in response to stable cues to become a habit, the participants will be accompanied by the same PV during the study [21].

#### Peer volunteers

We are collaborating with the Institute of Active Ageing (IAA), which is hosted by the Faculty of Health and Social Sciences of the Hong Kong Polytechnic University. The IAA is dedicated to delivering innovative educational programs to promote active aging and the view of older adults as important contributors to society. It provides many opportunities for older adults to contribute to society [22]. PVs will be recruited from a pool of regular members in the IAA. The IAA staff will announce and promote this study to those members. Those members who are interested will be invited to register and participate in the study. A total of 30 will be recruited. It is assumed that older adults who apply for membership in the IAA are active and willing to become involved in group activities. IAA members who express an interest will attend a selection interview. The selection criteria for the PVs include: ≥50 years old, retired, has a positive attitude towards aging and peer support, committed to completing two training workshops, passes the knowledge test, is able to demonstrate the use of a booklet among peers after the training workshops to the satisfaction of the research team, and is willing to provide services after the training. Retirees will be chosen, as their schedule is assumed to be more flexible, allowing them to arrange the time to volunteer.

### Training for peer volunteers

Prospective PVs will attend two ***training workshops*** prior to the 8-week group-based IPMP. They will be informed on the procedure of the program, the correct way to implement a variety of exercises and multisensory therapy, communication skills, and personal safety. Exercises and examples of multisensory therapy will be demonstrated to them. They will also be instructed to pair up with one another and to role play. The trainers will be the Principal Investigator and the research team. The content of the training will be validated by the Principal Investigator and the research team.

### Roles of peer volunteers

The PVs will work with the research team to develop the ‘I can do it’ booklet. They will contribute ideas for the content of the booklet. This will help to increase their knowledge, satisfaction, and compliance with the program. The PVs will then act as facilitators in the 8-week IPMP. Session 1 of each week will include 30 minutes of interactive teaching and sharing, 20 minutes of exercise, and 10 minutes of making entries in and reviewing the ‘I can do it’ booklet. The PVs will visit the nursing home with the research assistant in session 1 and assist in guiding the participants to do exercises and carry out the activities. Within the same week, session 2, which includes 30 minutes of revision, 20 minutes of exercise, and 10 minutes of entry and reviewing the ‘I can do it’ booklet, will be conducted by the PVs only. The content that the participants covered in the previous session will be revised and any remaining arts and crafts activities will be continued. It is expected that the participants will develop a habit of exercising and using non-pharmacological methods of pain relief after regular reminders and accompaniment by the PVs. The PVs will also be responsible for following up on the participants after the 8-week IPMP by visiting them weekly during weeks 9 to 12 to remind them to do their exercises and apply non-pharmacological strategies.

### Support for peer volunteers

The research team will be present in session 1 of each week and will share information and teaching experiences with the PVs. During session 2 of the first two weeks, the research team will observe the PVs facilitate the sessions and provide feedback at the end of the session. Any difficulties that are encountered will be discussed, mutual support and encouragement will be provided, and recognition of the achievements of the PV will be given to enhance their satisfaction. PVs can also call the research team for prompt support when necessary. A guidebook for PVs will also be developed as a reminder for the PVs.

### Measures of the quality of the peer volunteers

Guidebooks will be given to the PVs as guidance for practice so that they will be able to work independently according to the standard protocol. In assessing the PVs in terms of their adherence to the study protocol, the PVs will be required to pass a knowledge test and demonstrate the use of the booklet among their peers to the satisfaction of the research team after the training session and before they facilitate the sessions. The PVs will also be required to participate in session 1 of each week. A monitoring system will be put in place to allow the research team to observe the PVs during the first two weeks and to discuss with the PVs any issues that might arise.

Eligible participants and peer volunteers will be given written information about the study and required to return a consent form.

### Ethics committee

This project has already obtained ethical approval from the Departmental Research Committee of The Hong Kong Polytechnic University (Reference Number: HSEARS20140124002).

### Study design

#### Procedure

**Figure 1:**
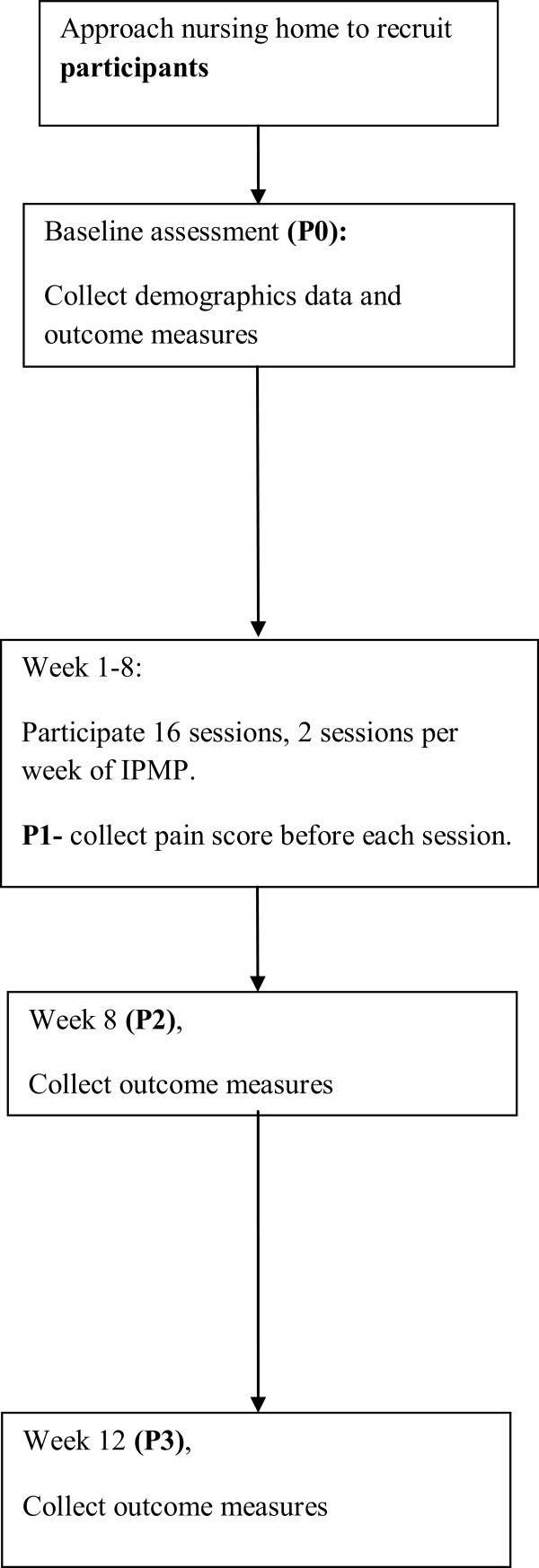
Research study design for participants.

**Figure 2:**
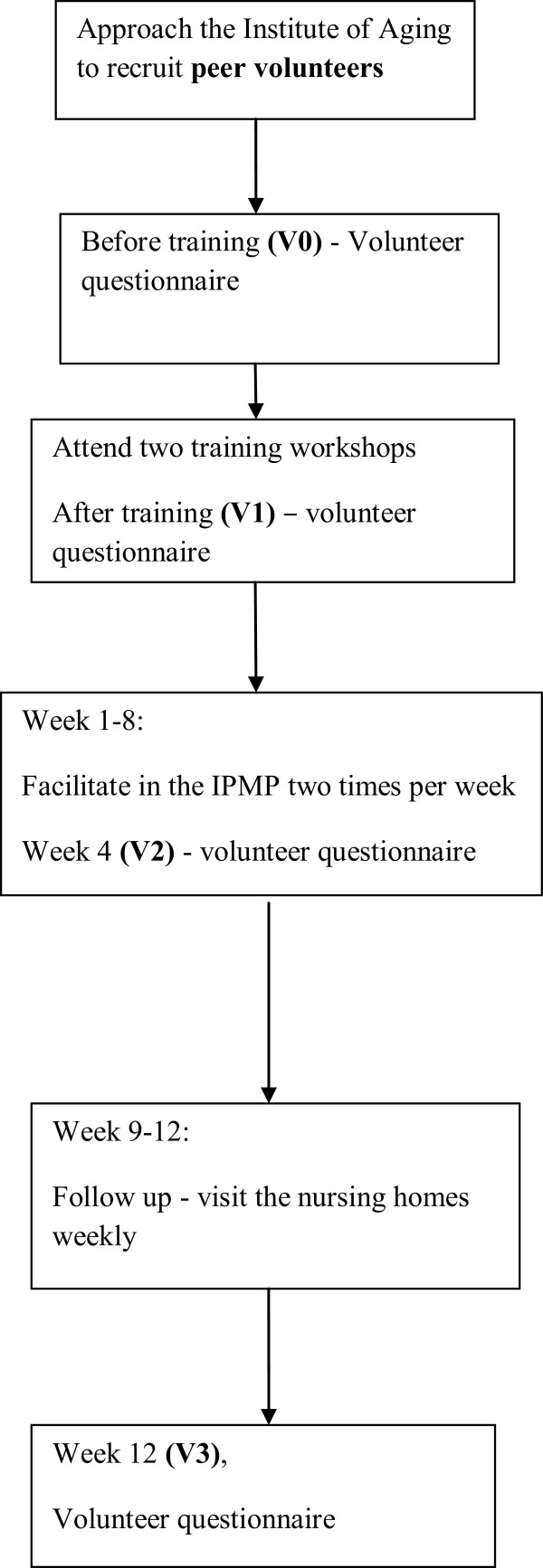
Research study design for peer volunteers.

As in Figure 1, the following data will be collected on the participants at P0 (baseline, that is, before the IPMP): demographic data (age, gender, marital status, medical history, educational level), pain score, functional mobility, physical activity, loneliness level, happiness level, and use of non-pharmacological pain management methods. An 8-week group-based IPMP during weeks 1 to 8 will then be offered. Each session will involve a group of 15 participants. To detect the dose-response relationship, pain scores will be collected at the beginning of each session from weeks 1 to 8 (P1). At week 8 (P2) and week 12 (P3), the following data will be collected: pain score, functional mobility, physical activity, loneliness level, happiness level, and use of non-pharmacological methods to relieve pain.As in Figure 2, PVs will complete the questionnaire for volunteers at four time points, that is, before the training (V0), after the training (V1), at week 4 (V2), and at week 12 (V3).

#### Preparation of the booklet and relevant materials

The booklet and other relevant materials will be prepared as indicated below:

1. ‘I can do it’ booklet. A booklet will be given to each participant at the beginning of the IPMP, and they will be asked to bring this booklet to all sessions. The booklet summarizes the key points of pain management strategies learned in the program and demonstrates the steps of various types of exercises. This booklet of each participant will be stamped every time they participate in a session. Outside the classroom, the booklet will also be used to record their participation in exercises or their application of other non-pharmacological methods. The booklet will be validated by the research team.

2. Materials for the IPMP. Various class materials will be delivered in the IPMP. Materials include music CDs for participants to listen to when they practice deep breathing; herbal tea to stimulate their taste buds; and scissors, glue, origami paper, and colorful art materials for the arts and crafts therapy to facilitate fine motor activities. All of the materials will be kept in the nursing home for the PVs to use until the end of the study.

3. Guidebook for peer volunteers. A guidebook will be developed for the PVs to ensure that the procedure and content of each session are standardized. Safety and privacy issues will also be mentioned in the guidebook. Links to webpages on pain management strategies will be printed for their reference. The guidebook will also include guidelines on how to use the ‘I can do it’ booklet. The guidebook will be validated by the research team, as well as by an advanced practice nurse, consultant, and physiotherapist from the Pain Clinic.

#### Evidence-based practice: Integrated pain management program (IPMP)

The participants will be given an 8-week group-based IPMP supplemented with PVs. Table 1 shows the details of the IPMP. We will follow the study design of a previous study [7]. There will be 16 sessions, with two 1-hour sessions each week.

**Table 1:** Integrated pain management program (IPMP)

**Week**	**Session 1**			**Session 2**		
	**(research assistant and peer volunteers)**			**(peer volunteers only)**		
	Interactive teaching and sharing	Exercise	Entry	Revision	Exercise	Entry
		(20 minutes)	(10 minutes)	(30 minutes)	(20 minutes)	(10 minutes)
	(30 minutes)					
1	Introduction on pain	Deep breathing	Research assistant and PVs will work together with the participants to make entries in the ‘I can do it’ booklet on the activity of the day.	PVs will use the ‘I can do it’ booklet to review the content taught in the previous session and continue any remaining arts and crafts activities.	Deep breathing	PVs will work together with the participants to make entries into the ‘I can do it’ booklet on the activity of the day.
	Impact of pain					
	Drug therapy					
2	Hot pad and cold pad	Relaxation exercise			Relaxation exercise	
3	Music therapy	Warm-up exercise			Warm-up exercise	
4	Massage	Shoulder and neck exercise			Shoulder and neck exercise	
5	Use of five sensations as nondrug therapies: making a photo album	Lower arm exercise			Lower arm exercise	
6	Use of five sensations as nondrug therapies: making a bag of dried flowers	Towel dancing			Towel dancing	
7	Use of five sensations as nondrug therapies: tasting of tea	Towel dancing			Towel dancing	
8	Wrapped up	Whole body exercise			Whole body exercise	

Session 1 of each week aims to empower the older adults with knowledge of how to manage pain and to demonstrate the procedure of the selected exercise. The research assistant (RA) will lead the sessions and the PVs will help the RA to guide the participants in doing exercises and assist them in carrying out the activities. Session 1 includes:

1. 30 minutes of interactive teaching and sharing. The contents include an introduction on pain, the impact of pain, drug therapy, five sensation therapy (touch, smell, taste, hearing, and vision), making a photo album and bags of dried flowers, and a tea tasting.

2. 20 minutes of exercise. Specific exercises for the shoulder, neck, arms, and the whole body, and balancing and towel dancing will be taught.

3. 10 minutes of making entries and reviewing the ‘I can do it’ booklet.

Session 2 of each week will be led by the PVs only. It includes:

1. 30 minutes of reviewing what the participants learned in the previous session and continuing any remaining arts and crafts activities;

2. 20 minutes of exercise to help them to develop it as a habit; and

3. 10 minutes of making entries and reviewing the ‘I can do it’ booklet.

### Outcome assessment

#### Primary outcome

The primary outcome measure is pain intensity, which will be assessed using an 11-point numeric rating scale (NRS). The NRS is a line marked in equal segments from 0 (no pain) to 10 (worst possible pain). The NRS has been shown to be a reliable and valid measure of pain intensity and pain distress in older patients with persistent pain [23].

#### Secondary outcomes

Functional mobility will be measured by Timed Up and Go Test [24]. The participants will be required to walk a distance of three meters, turn, walk back to the chair, and sit down. It is a short test of basic mobility skills. Participants who can finish the test within ten seconds will be interpreted as having normal mobility. Those who can do it in 11 to 20 seconds will be interpreted as having good mobility and not requiring a gait aid. Those who require 21 to 30 seconds to complete the test will be regarded as having problems with their gait and requiring a gait aid. The participants will be instructed to do the test with their usual footwear. There will be no time limit for the test. The RA will stay beside the participants during the test for timing and safety purposes. The test-retest reliability of this test is 0.97 [25]. A practice trial will be given and the test will be performed once with time recorded [26].

Physical activity will be assessed from the notes in the ‘I can do it’ booklet as well as from the results of the Global Physical Activity Questionnaire (GPAQ) developed by the World Health Organization. The GPAQ consists of several components of physical activity including intensity, duration, and frequency. It has been shown to be valid and reliable, and also adaptable to the incorporation of cultural differences [27].

The use of non-pharmacological methods will be recorded in the ‘I can do it’ booklet. The frequency of the use of non-pharmacological methods will be counted as the number of times per week without considering duration, as we simply want to provide tools for the older adults to manage their pain. The participants will be asked to tick the boxes for the corresponding pain-relieving method whenever they apply (for example, listening to music, deep breathing, and smell stimulation).

The loneliness level of the participants will be measured using the Loneliness Scale [28]. The scale consists of 20 items to assess the participants’ perception of loneliness and social isolation using a 4-point Likert scale (1 = never, 2 = seldom, 3 = sometimes, 4 = always). The total possible scores range from 20 to 80, with higher scores indicate greater loneliness. The Chinese version will be used with a Cronbach’s alpha of 0.9 [29].

The happiness level of the participants will be assessed by the Chinese version of the subjective happiness scale [30]. It consists of four items rated on a 7-point Likert scale. The total scores range from 4 to 28, with higher scores indicate higher subjective happiness. The Cronbach’s alpha is 0.79 to 0.94. The test-retest reliability ranges from 0.55 to 0.90 [30].

The questionnaire for volunteers, which contains open-ended and closed questions, will be completed by the PVs. Closed questions include those on their self-efficacy in learning and implementing the IPMP, self-rated knowledge of pain management; rating of the training, satisfaction with the program, and skills in delivering it. Examples of open-ended questions include attitudes and prior experiences of involvement in voluntary programs for older adults, expectations of the program, perceptions of various aspects of the program, and perceptions of barriers to and facilitators of the program.

#### Data analysis

The IBM-SPBB version 20 will be used to perform a statistical analysis. Descriptive statistics (frequency, %), mean (standard deviation)) will be used to describe the demographic data of the participants.

To determine the dose-response relationship of this intervention program on pain, a generalized estimating equation with quadratic and higher order terms will be used. To examine the intervention effect, a t-test will be used to compare the mean pain intensity, functional mobility, physical activity, loneliness and happiness levels, and use of non-pharmacological methods collected at four time points, that is, between P0 and P1, P1 and P2, P1 and P3 (for pain only), and between P0 and P2, P0 and P3, and P2 and P3 (for other variables). The Kolmogorov-Smirnov normality test will be used to examine the normality of the outcome variables. If the data do not follow a normal distribution, we will use the Wilcoxon Signed-rank test and the Mann-Whitney U test for within-group and between-group comparisons, respectively. A *P* value of <0.05 will be considered statistically significant. An intention-to-treat analysis will be conducted for any missing data. All data from the ‘I can do it’ booklet will be gathered and used to calculate the participation rate.

The questionnaires for the volunteers will be compared using the McNemar test and a pair sample t-test for quantitative data. Qualitative data will be analyzed using content analysis [31]. Sentences or parts of sentences will be identified as meaningful units in each questionnaire. The meaningful units will be categorized into themes related to the perception of program. The themes will be compared and discussed to establish the credibility of the interpretations.

## Discussion

The 8-week group-based IPMP has already been validated and published, and its effectiveness has been established. Interventions that include some type of social support have great potential to increase participation and adherence, raising the question of whether the effects of an IPMP can be extended with the use of PVs. This study is aimed at evaluating the feasibility of using PVs in an IPMP for Hong Kong older adults with chronic pain.

This pilot study helps older adults to control their pain by providing a PV to encourage and accompany them. The limitations of this study should be noted. As it is only a pilot study lasting 12 weeks, long-term changes in the outcome measures cannot be evaluated. Moreover, there may be a ‘learning effect’, which will familiarize the participants with measuring instrument in the study as there will be a repeated measure of pain intensity in the beginning of all 16 training sessions. This study provides evidence for future, larger studies that utilize a randomized controlled design and a possibly longer follow-up time to adjust this ‘learning effect’ and to investigate whether PVs should be integrated in programs for older adults with chronic pain. This will help to promote health among older adults in the long term.

## Trial status

This study is currently in the process of recruiting participants. The study is expected to start in March 2014.

## Abbreviations

GPAQ: global physical activity questionnaire; IAA: Institute of Active Ageing; IPMP: integrated pain management program; MVPA: moderate-to-vigorous physical activity; NRS: numeric rating scale; PEP: physical exercise program; PVs: peer volunteers; RA: research assistant.

## Competing interests

The authors declare that they have no competing interests.

## Authors’ contribution

MMYT is the overall principal investigator of the study, and the person who designed the study, arranged and participated in meetings with the staff of the Institute of Active Ageing, designed the content for training the volunteers, and participated in designing the manuscript as well as revising it for important intellectual content. PHL participated in designing the study, helped in the statistical analysis, and revised the manuscript for important intellectual content. SMN participated in designing the study, designed the content for training the volunteers, provided advice on the exercise component of the program, and revised the manuscript for important intellectual content. BKTW participated in designing the study, helped to recruit and train the volunteers, provided advice and helped to recruit the peer volunteers in the IAA, and revised the manuscript for important intellectual content. SSYY participated in designing the study and drafted the manuscript. All of the authors read and approved the final manuscript.
